# Textbook of Radiation Oncology

**DOI:** 10.1038/sj.bjc.6602377

**Published:** 2005-02-22

**Authors:** T Prior

**Affiliations:** 1 University College London Hospitals, London, UK

The second edition of this textbook, published 6 years after the first, is edited by two eminent radiation oncologists from the Memorial Sloan-Kettering Cancer Centre, New York, and the University of California, San Francisco. The editors state that their aim was to use contributors that had connections with these institutions in order to present a uniform treatment policy to ‘teach the reader how to treat for cure’. With only four of the 123 contributors being based at European institutions, the reader has to be prepared for a North American bias in the presentation of treatment policies and evidence for them. Having said that, this book contains a wealth of information for any radiation oncologist, from a first year trainee upwards.

In the past 6 years, radiotherapy technique has evolved rapidly and this book does a fine job in documenting these changes, aided by 71 colour plates to illustrate the text. As the editors point out, gone are the days of simply memorising ‘classical’ ports; in their place is a complex process of target identification and definition, treatment delivery and verification. 3D conformal radiotherapy and intensity-modulated radiotherapy (IMRT), a way of conforming dose more accurately to the target volume, is now in widespread use and a particular emphasis is placed on this throughout the book.

The book is divided into four sections: radiation physics and biology, imaging, clinical radiation oncology and emerging radiation modalities. It is well organised with the general principles behind radiotherapy being well explained and illustrated. I particularly enjoyed Kian Ang's ‘fractionation in clinical practice’, which gave a clear overview of the merits of different fractionation regimens – a perennial ‘hot topic’ in practice. However, this was where I found the only publishing error apparent to me – an important omission of text pertaining to the late effects of hybrid accelerated fractionation regimens. The overview on chemotherapy and radiotherapy also offers a well-written update on the evidence for chemoradiation and the biological mechanisms of interaction of the two modalities of treatment. The chapter devoted to immobilisation and simulation is excellent, with a clear and logical survey of the principles which will be useful to anyone starting out in radiotherapy.

One of the main strengths of the book is the emphasis on imaging. In recent years, radiation oncologists have been able to use many imaging modalities to help identify the tumour or tumour bed they will be delivering the radiotherapy to. The images acquired from a patient form the basis on which the radiotherapy plan is produced and target definition has developed from a two-dimensional concept to a 3D one. As a result, radiation oncologists have had to redefine what constitutes the ‘target’, as well as having to identify and outline the target with ever-increasing accuracy. Reflecting the increasing emphasis on interpreting images for radiotherapy planning, there is a very useful overview of imaging in oncology as well as a whole section devoted to imaging, divided up into site-specific chapters. This will be most useful to anyone with an interest in oncology. Novel imaging approaches such as multimodality image registration and fusion are also covered in depth, with some excellent supporting colour plates and illustrations.

The site-specific chapters in the section devoted to clinical radiation oncology follow a standard layout for most chapters, with sections on incidence, anatomy, pathology, clinical presentation, routes of spread, diagnostic studies, staging, prognostic factors and standard therapeutic approaches. The editors are true to their word and there is a definite emphasis on first-line treatment. There is, relatively, little information given in the treatment of recurrent disease and salvage treatment. While this was the remit of the book, many patients do relapse despite excellent treatment, and many oncologists are faced with this situation daily. Retreatment is a controversial area and a chapter giving an overview on the challenges an oncologist faces in this position would, I'm sure, have been welcomed.

The editors have stressed the importance of modes of spread allowing decisions on target volumes to be made more rationally, which is particularly important with IMRT planning. Modern immobilisation techniques, precise tumour targeting, radiotherapy simulation, treatment delivery and verification are all looked at in depth for each tumour site. Normal tissue reactions as well as late effects are documented, making this clinical section easy to read and informative. There is an excellent chapter on the treatment of benign disease, which adds to the wealth of knowledge presented in the treatment of malignant disease.

The final section on emerging radiation modalities gives an interesting overview on techniques which are yet to become widespread in use. They offer an insight into particle radiation therapy, hyperthermia, photodynamic therapy, tumour-targeted radioisotope therapy and extracranial stereotactic radioablation.

In today's multidisciplinary era, it is unusual to find a textbook with such a focus on decision-making and treatment from the radiation oncologist's point of view. I think this textbook will therefore be enjoyed by any radiation oncologist either in training or as an established practitioner, as well as anyone else who is involved in radiation oncology treatment planning and delivery. I would also expect that medical oncologists would also find this a useful book to have in their library. In my opinion, the editors have achieved what they set out to do.

